# Radiotherapy services in Brazil: current scenario, challenges, and proposals for solutions

**DOI:** 10.1590/0102-311XEN127324

**Published:** 2025-09-01

**Authors:** Raquel Guimarães Domingos da Silva, Claudia Affonso Silva Araujo

**Affiliations:** 1 Instituto Nacional de Câncer, Rio de Janeiro, Brasil.; 2 Instituto Coppead de Administração, Universidade Federal do Rio de Janeiro, Rio de Janeiro, Brasil.

**Keywords:** Neoplasms, Access to Health Services, Low and Middle Income Countries, Radiotherapy, Delphi Method, Neoplasias, Acesso a Serviços de Saúde, Países de Baixa e Média Renda, Radioterapia, Método Delphi, Neoplasias, Acceso a los Servicios de Salud, Países de Bajos y Medianos Ingresos, Radioterapia, Método Delphi

## Abstract

This study aimed to provide a consensus-based short list of barriers and challenges to establish accessible radiotherapy services in Brazil and to discuss the managerial actions proposed to reduce the waiting time to initiate radiotherapy treatments. An e-Delphi study was made with no direct interaction between respondents. Virtual surveys were sent to physicians who had expertise in radiotherapy. A baseline list of 15 previously published barriers to radiotherapy access in low- and middle-income countries was put in topics. Participants had to rate the priority of including each of the 15 topics in future governmental interventions by using a 5-point Likert scale. Average scores for each topic were calculated and expressed as percentages. Consensus was achieved if the topic obtained a score of > 70% agreement among the participants that rated it as very high or high priority, plus being in the top five in the ranking list of importance. Four topics reached consensus. Two topics were related to costs (resources, funding models, and financial stability), one to policy environment (political instability), and one to poverty levels and planning distribution of technology. Such results form the basis for an action plan and the comprehensive priority topics should be considered in the efforts to provide better access to radiotherapy services.

## Resumo

Este estudo buscou fornecer uma pequena lista consensual de barreiras e desafios para o estabelecimento de serviços de radioterapia acessíveis no Brasil e discutir as ações gerenciais que foram propostas para reduzir o tempo de espera para o início desses tratamentos. Pesquisas virtuais foram enviadas a médicos com experiência em radioterapia nesse estudo e-Delphi sem interação direta entre os entrevistados. Uma lista básica de 15 barreiras já publicadas ao acesso à radioterapia em países de baixa e média renda foi colocada em tópicos. Os participantes tiveram que estabelecer a prioridade com que incluiriam cada um dos 15 tópicos em futuras intervenções governamentais usando uma escala Likert de cinco pontos. As pontuações médias para cada tópico foram calculadas e expressas em porcentagens. Considerou-se em consenso os tópicos com uma pontuação de > 70% de concordância entre os participantes que os classificaram como tendo alta ou muito alta prioridade e uma posição entre os cinco primeiros itens na lista de classificação de importância. No total, quatro tópicos obtiveram consenso. No mais, dois tópicos se relacionavam a custos (recursos, modelos de financiamento e estabilidade financeira), um ao ambiente político (instabilidade política) e um aos níveis de pobreza e planejamento da distribuição de tecnologia. Tais resultados formam a base para um plano de ação em que os tópicos prioritários abrangentes devem ser considerados nos esforços para fornecer melhor acesso a serviços de radioterapia.

## Resumen

Este estudio tuvo como objetivo proporcionar una lista corta basada en consenso acerca de las barreras y desafíos para establecer servicios de radioterapia accesibles en Brasil y discutir las acciones de gestión propuestas para reducir el tiempo de espera para iniciar los tratamientos de radioterapia. Se realizó un estudio e-Delphi sin interacción directa entre los encuestados. Se enviaron encuestas virtuales a médicos con experiencia en radioterapia. Se incluyó en los temas una lista de referencia de 15 barreras para el acceso a la radioterapia publicadas anteriormente en países de ingresos bajos y medianos. Los participantes tuvieron que calificar la prioridad de incluir cada uno de los 15 temas en futuras intervenciones gubernamentales utilizando una escala Likert de cinco puntos. Se calcularon las puntuaciones medias de cada tema, las cuales se expresaron en porcentajes. El consenso se logró si el tema obtuvo un puntaje de > 70% de acuerdo entre los participantes que lo calificaron como de muy alta o alta prioridad, además de estar entre los cinco primeros en la lista de clasificación de importancia. Cuatro temas alcanzaron consenso. Dos temas estaban relacionados con los costos (recursos, modelos de financiamiento y estabilidad financiera), uno con el entorno político (inestabilidad política) y otro con los niveles de pobreza y la planificación de la distribución de tecnología. Estos resultados constituyen la base de un plan de acción y los temas prioritarios globales deben ser considerados en los esfuerzos por proporcionar un mejor acceso a los servicios de radioterapia.

## Introduction

The World Health Organization (WHO) has identified neoplasms as the cause of one in every six deaths globally ^1^. The latest global data from 2018 report 18 million new cancer cases worldwide ^2^, with projections indicating a rise to 24.6 million by 2030 and 29.6 million by 2040, especially in low- and middle-income countries (LMICs) ^3^. As there are advancements against infectious and cardiovascular diseases, cancer is becoming a major global health issue ^4^.

Radiotherapy is essential in cancer treatment and is known for its cost-effectiveness in treatment and palliation. About two-thirds of cancer patients will need radiotherapy at some stage ^5^, and it is also used in some benign conditions ^6^. However, radiotherapy access and equipment availability, such as linear accelerators (LINAC), significantly vary, often mirroring a country’s socioeconomic status ^7^. Consequently, the increase in cancer cases, demographic shifts, and more radiotherapy-inclusive treatment protocols may lead to extended waiting times ^8^. The negative impact of these delays on survival rates has been well-established, emphasizing the need for timely radiotherapy access ^9^ ^,^ ^10^ ^,^ ^11^.

Efforts to overcome barriers to cancer care have been mainly conducted in high-income countries ^12^, leaving LMICs facing a critical shortage of radiotherapy services and a lack of epidemiological data for capacity planning and building ^13^. Brand et al. ^14^ highlight significantly longer waiting time for cancer treatment in LMICs (median delay of 6.5 months) compared to wealthier regions, with low health literacy often shown as a significant barrier to early diagnosis.

In Brazil, which is marked by great inequalities in healthcare access, disparities are influenced by factors such as age, geographical location, disease stage, race, and referral source. Also, a significant mismatch exists between the distribution of cancer cases and the availability of LINAC for radiotherapy ^15^.

A 2011 Brazilian Federal Audit Court report exposed severe deficiencies in radiotherapy services, showing an average wait of 113.4 days from diagnosis to the beginning of the treatment under the Brazilian Unified National Health System (SUS, acronym in Portuguese). SUS serves most of the Brazilian population, far from meeting the Brazilian National Policy on Oncologic Care’s mandate of starting treatment within 60 days post-diagnosis ^16^. Still, by 2015, it was estimated that over 100,000 patients lacked access to radiotherapy in Brazil ^17^. In 2019, the country had 409 therapy machines, of which 100 (24%) were dedicated to SUS, 93 (23%) to the private sector, and 216 (53%) attending both ^16^. In 2030, 639,994 new cancer cases are expected in Brazil (a 41% increase compared to 2018), requiring 332,797 radiotherapy treatments and 530 therapy machines ^5^ ^,^ ^16^.

In a prior study, Donkor et al. ^18^ showed multiple obstacles to establish enduring, high-quality radiotherapy services in LMICs, yet there is no consensus on which are most critical. This study seeks to create a consensus-based list of barriers to radiotherapy access in Brazil, aiming to prompt effective interventions to improve access to radiation oncology care and reduce delays on treatment initiation.

## Methods

This study used the electronic Delphi method (e-Delphi) to reach a consensus on barriers to radiotherapy access in Brazil, facilitating the process without direct interaction among respondents. The e-Delphi survey effectively ensures a globally representative panel ^19^. Participants, which were identified as “experts”, anonymously answered questionnaires, and their answers were summarized and shared with the group. This approach enables the participants to refine their opinions and change their responses after considering the group’s collective insights, a lacking feature in traditional single-intervention qualitative methods ^20^ ^,^ ^21^. The Delphi technique is particularly effective in identifying experts’ implicit knowledge regarding barriers to cancer care, capturing collective wisdom that might not be easily expressed and making communication accessible and results interpreted quickly ^22^ ^,^ ^23^.

This study was carried out from January to March 2024 and was approved by the Human Research Ethics Committee of the Federal University of Rio de Janeiro (approval n. 69106623.9.0000.5582). Participants provided their signatures on the informed consent form before completing the survey.

### Participants and setting

The participants were experts in managing radiation oncology policies to reduce the waiting time for radiotherapy treatment once it is indicated. A diverse panel was assembled to ensure a broader perspective and generalizability of the consensus. This panel, which was compiled from the Brazilian Society of Radiation Oncology technical meetings archives, covers experts from different backgrounds and Brazilian regions, thereby representing a comprehensive view of the issue.

As there is no standardized approach to establish the size of a Delphi study panel ^24^, the authors assembled 70 members, balancing between creating definitive outcomes and managing the challenges associated with larger groups ^20^. An invitation email was sent to all members, outlining the study goals, the method to select barriers, participant expectations, and an informed consent form.

### The questionnaire

A list of 15 barriers (Box 1) was derived from Donkor et al. ^18^, who conducted an exploratory-descriptive qualitative study with semi-structured interviews among 17 individuals (oncologists, medical physicists, radiation therapists, and administrative staff) involved in LMIC radiotherapy services, requiring a minimum of two years of experience. The experts had to agree or disagree with the themes and subthemes proposed by Donkor et al. ^18^ and suggest new ideas and contributions to establish accessible radiotherapy services in Brazil.

**Box 1:** Barriers to establish and sustain radiotherapy services in low- and middle-income countries (LMICs).

CATEGORY	ELEMENT
Policy environment	Political impasses
	Bureaucratic corruption
	Lack of political leadership continuity
	Competing political demands on scarce resources
	Policymakers’ negative misperception about radiotherapy
Advocacy	Feelings of powerless to influence decision
	Lack of coordinated advocacy effort
Radiation safety	Nonmember State of the IAEA
	Lack of legal and regulatory framework
Country-wide radiotherapy implementation plan	Poor planning
	Lack of reliable epidemiological data
Identifying a funding model	Lack of a comprehensive and reliable line-item budget
	Poor mobilization and allocation of financial resources
Building the facility and purchasing the right machinery	Lack of local radiotherapy expertise
	Poor stakeholder engagement
	Lack of a competent project manager
	Lack of negotiating power of LMICs
	Lack of coordination and communication among relevant stakeholders
	Lack of synchronization of different activities
Building the radiotherapy workforce	Absence of suitable trained workforce
	Lack of sufficiently qualified teachers and mentors
	Lack of appropriate incentives
	High degree of brain drains after overseas education and training
	Lack of staff succession plan
Gaining and maintaining financial stability	Lack of financial resources to support service operation
	Financial hardship to patients and their families
Regular maintenance	Lack of a long-term service contract
	Poor spare parts supply and distribution
	Expensive spare parts
	Lack of qualified engineers
Building research culture and infrastructure	Lack of research capacity in radiotherapy
Good governance and management	Lack of clear roles and responsibilities
Improving patient outcomes	Lack of trust based on information and communication processes
	Lack of needed social support services
	Limited access to evidence-based clinical guidelines
Catalyzing comprehensive care and support service	Inability to plan and prioritize integration
	Expensive process for governments
A multidisciplinary approach to care	Differences between professional perspectives
	Lack of understanding the role of radiotherapy
Information and communication technologies	Inadequate training and assistance for users

IAEA: International Atomic Energy Agency.

Source: based on Donkor et al. ^18^.

### Definition of consensus

The Delphi study sought consensus by gathering informed opinions on two key aspects: (1) the importance of enhancing public access to radiotherapy services and (2) the impact of these topics on guiding public policies.

The experts were tasked with two actions for each of the 15 preliminary topics to prioritize them: (1) Evaluate the priority level using a Likert-type scale (very high, high, average, low, very low), in which the percentage of participants assigning a “very high” or “high” priority was calculated for each topic; (2) Order the topics based on priority (mean ranking score, in which a lower mean indicates a higher priority). If a topic had a “very high/high” priority from at least 70% of participants and ranked among the top five based on mean scores, it reached a final consensus.

Inclusion criteria for topics in subsequent rounds required a minimum 70% consensus or a mean Likert scale score above 4 in the first round, except for topics suggested by respondents. While the median threshold for consensus in Delphi studies is often around 75%, setting a consensus level at 70% has been effectively used and accepted in various studies. This flexibility enables researchers to tailor the consensus level to the aims of their study ^25^ ^,^ ^26^ ^,^ ^27^.

### Procedures

The survey was distributed, and the answers were collected via Google Forms (https://docs.google.com/forms). The participants were receiving periodic reminders to ensure engagement, a sense of ownership, and collaboration. This approach encouraged active participation in each survey round ^28^.

Anonymity of the participants was maintained throughout the process to prevent any expert from unduly influencing the consensus ^29^. One of the researchers gathered and analyzed the data, compiling a comprehensive summary report, omitting personal details, and providing controlled feedback. This method adhered to the established consensus criteria across all rounds, using repetitive and interactive surveys to facilitate discussion.

The initial procedure focused on gathering demographic and professional information from participants, including gender, date of birth, geographic region of practice, years of experience, current job position, and whether their primary work setting was in the public or private sector. The first and second rounds delved into the goals of the study, employing a Likert scale for evaluation and a ranking system to prioritize answers, respectively. In the first round of the Delphi process, experts assessed the importance of the impact of each item on radiotherapy access in Brazil using a 5-point Likert scale, ranging from 1 (“very low relevance”) to 5 (“very high relevance”). Additionally, they were invited to identify any other barriers that could affect radiotherapy access in Brazil, incorporating the suggestions into the next round. The recommended barriers and challenges were integrated in the second round, resulting in a revised version with eight constructs. Participants were then asked to prioritize these items, ranking them from 1 (highest priority) to 8.

The Delphi rounds occurred two weeks apart. Before each round, the authors sent to the participants a document containing pertinent information - such as a summary of the results of the first round before the second round - and a link to the online survey.

## Results

**Figure 1:**
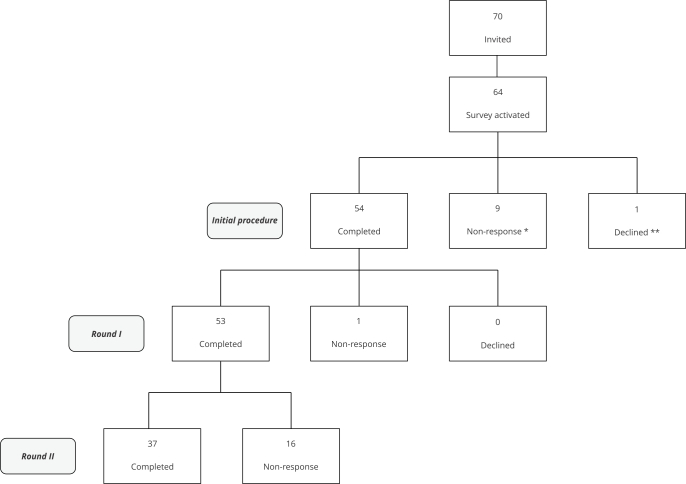
Recruitment and completion of the Delphi rounds.

Out of the 70 individuals invited, 53 answered to the first round (75.7%) and 37 to the second round (52.8%), comprising the final sample (Figure 1).

* Participants who did not start rounds I or II surveys; ** Participants who declined to provide consent or requested to withdraw from further participation.

The radiation oncologists bring diverse professional backgrounds to the study. They reside across 10 states in Brazil, each with over 10 years of experience in the field, ensuring a comprehensive perspective (Table 1).

**Table 1:** Characteristics of the Delphi respondents (n = 54).

Characteristics	n (%)
Sex	
Male	32 (59)
Female	22 (41)
Age (years)	
30-39	13 (24)
40-49	25 (46)
50-59	6 (11)
≥ 60	10 (18)
Region (State of Brazil)	
Bahia	3 (5)
Ceará	1 (2)
Pernambuco	2 (4)
Espírito Santo	2 (4)
Rio de Janeiro	19 (35)
São Paulo	13 (24)
Minas Gerais	4 (8)
Goiás	2 (2)
Paraná	1 (2)
Rio Grande do Sul	4 (8)
Federal District	3 (5)
Years of experience	
< 5	2 (4)
5-10	10 (18)
11-15	11 (20)
16-20	12 (22)
21-25	9 (17)
> 25	10 (18)
Head of Department	
Yes	27 (50)
No	27 (50)
Main job	
Public	21 (39)
Private	21 (39)
Philanthropic	12 (22)

In Round I, the experts identified six items as essential for establishing and maintaining timely access to radiotherapy services in Brazil: (1) Policy environment; (2) Nationwide implementation plan; (3) Identifying a funding model; (4) Building the facility and acquiring the right machinery, and (5) Achieving and maintaining financial stability, for garnering a “very high/high” priority from at least 70% of participants, and (6) Advocacy - the sense of being unable to influence the decisions of stakeholders, for achieving a mean Likert scale score of 4.22. Such issues qualified for the second round, along with two new suggestions by the participants: (1) Geographic distribution of machines - high concentration of equipment in big cities, mainly in South and Southeast regions of Brazil, and low availability of machines outside big centers, and (2) High duties to import machines - lack of tax exemptions to the private sector to import technology since they are not produced in Brazil (Table 2).

**Table 2:** Summary of the first round of the Delphi survey about barriers to promote access in radiotherapy services in Brazil (n = 53).

Category	Mean Likert scale	Degree of consensus *	Experts’ consensus **
Identifying a funding model	4.66	0.92	Included
Policy environment	4.60	0.92	Included
Advocacy	4.01	0.64	Included
Country-wide radiotherapy implementation plan	4.13	0.77	Included
Building the facility and purchasing the right machinery	4.13	0.75	Included
Gaining and maintaining financial stability	3.98	0.70	Included
Building the radiotherapy workforce	3.64	0.58	Excluded
Good governance (roles and responsibilities)	3.84	0.62	Excluded
Catalyzing comprehensive care and support service	3.69	0.62	Excluded
Regular maintenance	3.66	0.60	Excluded
Improving patient outcomes	3.58	0.43	Excluded
Multidisciplinary approach to care	3.54	0.47	Excluded
Building research culture and infrastructure	3.40	0.45	Excluded
Information and communication technologies	3.26	0.37	Excluded
Radiation safety	2.83	0.24	Excluded
Geographic allocation of the machines	NA	NA	Suggested by respondents/Included
High duties to import machinery	NA	NA	Suggested by respondents/Included

NA: not applicable.

* Agreement level by the participants on the category being very high/high impact;

** Category had to reach ≥ 70% consensus or to reach a mean Likert scale ≥ 4 to be included in the following round, except for categories respondents suggested.

In Round II, experts received a second questionnaire summarizing the group’s responses and the consensus level achieved. This procedure enabled the experts to reconsider their initial responses and adjust their rankings accordingly. They were then asked to prioritize the items from 1 to 8, in which one was the highest priority. The “policy environment” received the lowest priority, yet it still garnered an 80% consensus as being among the top five most significant obstacles to improve access to radiotherapy services (Table 3).

**Table 3:** Summary of the second round of the Delphi survey about barriers to promote access in radiotherapy services in Brazil (n = 37).

Category	Mean ranking score	Degree of consensus *	Final consensus **
Identifying a funding model	3.83	0.65	No
Policy environment	3.70	0.78	Yes
Advocacy	4.54	0.57	No
Country-wide radiotherapy implementation plan	3.48	0.73	Yes
Building the facility and purchasing the right machinery	4.05	0.73	Yes
Gaining and maintaining financial stability	2.81	0.81	Yes
Geographic allocation of the machines	4.02	0.67	NA
High duties to import machinery	4.05	0.65	NA

NA: not applicable.

* Agreement level by the participants on the category being among the top five in the ranking list;

** Category had to reach ≥ 70% consensus on the category of being of very high/high impact and to be among the top five in the ranking list (consensus in rounds I and II).

## Discussion

The Delphi study identified four primary barriers to accessing radiotherapy services in Brazil: the challenging policy environment, the need for a nationwide implementation plan, the crucial task of building the facility and acquiring the right machinery, and the pressing issue of gaining and maintaining financial stability.

According to the experts, the Brazilian “policy environment” is complex, marked by political impasse, bureaucratic corruption, frequent changes in political leadership, competing priorities for scarce resources, and misunderstandings among policymakers about the importance and functioning of radiotherapy. Such findings corroborate what is stated in the international literature. Abdel-Wahab et al. ^30^ highlight that, given the global state of radiotherapy, there is an urgent need for a wide-ranging competency inventory, which should detail the critical knowledge, skills, and necessary behaviors for safe, sustainable, and professional practice in radiation oncology.

Regarding a “nationwide implementation plan”, participants emphasized that 80% of patients in Brazil are served by the SUS radiotherapy services and that many patients must travel long distances to access health care because of the heterogeneous distribution of radiotherapy services. This issue is attributed to inadequate planning and a lack of epidemiological data. Worldwide access to radiotherapy varies significantly, often reflecting a country’s socioeconomic status and its resources. To improve access in settings with limited resources, Elmore et al. ^31^ argued that the substantial investments required to establish a radiotherapy program, alongside ongoing operational and maintenance demands, pose significant challenges for LMIC countries, particularly when weighed against other development priorities. However, even in countries with more machines, there still are challenges in effectively using radiotherapy, which include difficulties in implementing technology, ensuring safety and quality control, and the need for continuous education and training of healthcare personnel ^32^.

According to the experts, the issue of “building the facility and acquiring the right machinery” is pivotal to improve radiotherapy. Recent years have brought considerable progress in this field, significantly improving patient outcomes ^33^. The challenge is not merely in choosing new technologies or expanding access but in agreeing on the actual value of such improvements for radiotherapy. Careful investments in infrastructure and equipment to save limited budgets and resources are vital, which could further limit access and risk the financial sustainability of healthcare systems. Radiotherapy distinguishes itself by translating innovations into real-world benefits, notably in reducing toxicity and enhancing the quality of life for patients - outcomes that might only become apparent months or years after treatment ^15^.

“Gaining and maintaining financial stability” in Brazil is a significant concern, particularly in a country that lacks a detailed, reliable budget and inefficient financial management and allocation. The core issue is not the absence of resources but rather the misallocation of them. Radiotherapy problem in Brazil is not financial per se, as funds are indeed available. The issue lies in the management’s inability to effectively allocate these resources towards developing high-quality services. A lack of strategic planning and project implementation capabilities composes this inefficiency. Despite the availability of funds, radiotherapy improvement is hindered, leading to stagnation.

Additionally, the great expense of acquiring equipment, exacerbated by the national currency’s depreciation and high import duties, remains a huge barrier to expanding radiotherapy services. As proposed by Faroni et al. ^34^, a widely endorsed solution is to provide tax incentives or reductions for equipment purchased by the private sector if it also serves the public sector (public-private partnership). Also, the lack of protection of the internal market against imported products may be a reason why a radiotherapy device company (Varian Company) discontinued its factory services in Brazil.

In short, in Brazil, there is a great gap in access to cancer care, service availability, cost of treatment, and understanding of modern cancer care’s benefits. Such issues need to be tackled individually to enhance population outcomes. Creating a nationwide radiotherapy network demands huge investments and a highly-skilled workforce. Weltman & Marta ^17^ have pointed out that Brazil has been grappling with an economic crisis in recent years, leading to rising costs due to domestic inflation and currency depreciation, making expenses unpredictable. This economic climate hampers the replacement of outdated equipment, the development of new facilities, and the modernization of existing ones. Moreover, the issue of low compensation challenges attracting qualified professionals.

The country’s economic situation is delicate and seeking new resources is complex. Still, the specific needs of individual countries and regions should be considered to improve outcomes. An important facet of this challenge is the nationwide uneven distribution of radiotherapy devices. The installed capacity in the South and Southeast regions surpasses 60% of the demand. Conversely, it falls short in the North, Northeast, and Central-West regions, meeting less than 40% of the need ^33^. Assessing the needs and gaps in radiotherapy at a regional level is the first step in adequately planning the process, human resources, and infrastructure. Data-driven healthcare planning shows tremendous opportunities and many challenges in data collection and evaluation ^35^. Also, recommendations for the required number of radiotherapy resources often fail to fully consider the fast-paced developments in radiation therapy, including new indications, techniques, and fractionation schedules. Various costing models exist, using either time-driven activity-based costing methods or specific models to cancer types ^36^.

Furthermore, a proper number of trained radiotherapy professionals is essential for a sustainable and effective radiotherapy program. Yap et al. ^35^ ratified that a shortage of trained professionals is a serious hurdle to overcome in making radiotherapy accessible to patients with cancer. Hanna et al. ^37^ endorsed that in Brazil, it would be desirable to improve the quality of medical training in radiotherapy, including medical physicists, radioprotection supervisors, radiotherapy equipment operators, nurses, dosimetrists, engineers, and healthcare managers. Addressing this issue requires significant time and effort to develop and implement strategies in education and training that correspond to each country’s unique realities and challenges. Such educational initiatives are region-specific and somewhat isolated because of differences in standards, medical practice, and education ^35^. Trained professionals are critical for the sustainable growth of radiotherapy and should be a strategic consideration in any National Cancer Control plan. Prioritizing radiotherapy professionals’ initial education and ongoing training - including medical physicists, radiation therapists, and radiation oncologists - is vital. This includes supporting continued education to ensure they can update or broaden their expertise and skills ^38^.

When monitoring and enhancing access to radiotherapy services, it is essential to identify which barriers to focus on ^39^ ^,^ ^40^. This study highlights opportunities for collaborative efforts to support radiation oncology in LMIC. It also emphasizes the importance of advocacy and communication strategies in backing funding initiatives for worldwide radiotherapy development.

Box 2 shows the comprehensive recommendations to improve access to radiation oncology care and reduce treatment initiation delays in Brazil. The macro level should consider such recommendations, evaluating the conceptualization of health services ecosystems ^41^. We conducted a scoping review ^42^ in which the results indicate governments should implement large-scale programs to define and build new services infrastructure, train healthcare professionals and paraprofessionals, and invest in technology, especially in telecommunication, to overcome many on-site limitations in resources and expand access to health services. Such initiatives are critical in low and middle-income countries.

**Box 2:** Summary of the recommendations to improve access to radiation oncology care and reduce delays in treatment initiation in Brazil.

THEME	RECOMENDATION
Policy environment	To develop critical knowledge of politicians and positive perception of the importance of radiotherapy
	To promote political stability on planning continuity
	To favour political decision to invest in basic infrastructure
	To warrant strong political support on decisions and demands
Country-wide radiotherapy implementation plan	To provide safety and quality control
	To release a costed cancer control plan
	To provide accurate epidemiological data
	To enlarge allocation of machines
Building the facility and purchasing the right machinery	To share value cocreation of advancements in radiotherapy
	To stimulate access to experienced contractors
	To create a multidisciplinary implementation team
	To assure access to new technologies
Gaining and maintaining financial stability	To certificate clear financial mechanism for operational sustainability
	To plan correct allocation of investments
	To give supportive service operations to patients and their families
	To include tax incentives or reduction for private sector in public partnerships

## Conclusion

This study uncovers a consensus among experts on four major barriers to radiotherapy access in Brazil, providing a significant contribution to the field. Such barriers - including financial challenges, political hurdles, and the lack of epidemiological data - are crucial factors hindering the development of a comprehensive national radiotherapy strategy. The research also illuminates the intricacies of the Brazilian radiotherapy network, laying a solid groundwork for future cancer policy development to improve the current situation.

Regarding the research limitations, we acknowledge the critiques associated with the Delphi method, including its dependency on meticulous analysis of responses, the risk that answers may not be entirely independent, as we cannot assert that the participants communicated among themselves, and the possibility of bias in shaping opinions. Determining and achieving consensus also introduces a potential for bias in shaping opinions. We also recognize that our study scope, focusing primarily on actions for cancer treatment rather than prevention, may limit our ability to predict future trends accurately. The relatively sparse connections and interactions among respondents who operated within closed networks may have further constrained the diversity of insights and the potential for broader collaborative dynamics.

This study makes great contributions to public management. Insights from radiotherapy professionals and recommendations from regional and national analyses can serve as a beacon, guiding policymakers to effectively direct efforts and resources for maximum impact on improving access to radiotherapy. Furthermore, this research provides empirical evidence that can fuel policy debates and inform decision-making. For academics, this study enriches the existing literature on healthcare access and cancer treatment disparities in Brazil, offering valuable empirical data and insights. Such findings enrich academic discussion and enhance our understanding of the complexities surrounding radiotherapy access in diverse healthcare systems. Ultimately, this study contributes to societal well-being by guiding healthcare policy and practice improvements, particularly in access to radiotherapy treatment, and has the potential to directly benefit patients by ensuring more equitable and timely access to crucial treatments like radiotherapy.

We propose several avenues for future studies: (1) Investigating global issues such as demographic shifts towards an ageing population, innovations in treatment, and the impact of high-cost medications; (2) Delving deeper into the inequities in cancer treatment access within developing countries; (3) Fostering collaborations with key scholars in the global network, focusing on Latin America and LMICs.
